# Table of Meteorological Conditions in the City of Buffalo for September, 1854

**Published:** 1854-11

**Authors:** Sanford B. Hunt


ART. V.— *Table of Meteorology and Epidemics of Buffalo for September, 1854.* By SANFORD B. HUNT, M. D.

| Date. | In. of Barometer. | Rain. | | Amt. of Clouds. | Force and Direction of Winds. | Upper Station. Hygrometer. | | | Lower Station. Hygrometer | | | D'ths by Cholera. |
|---|---|---|---|---|---|---|---|---|---|---|---|---|
| | | Began. | Ended. | | | Temp. | Dew-p'nt. | Humid'y. | Temp. | Dew-p'nt. | Humid'y. | |
| 1 | 29.2 | | | 2 | 2. S.W. | 76. | 69. | 804 | 76. | 69. | 804 | 4 |
| 2 | 29.2 | | | 2 | 2. S.W. | 82. | 70.33 | 691 | 75. | 68. | 809 | 4 |
| 3 | 29.3 | | | 0 | 1. S.W. | 85. | 66.33 | 567 | 78. | 68.66 | 760 | 1 |
| 4 | 29.2 | | | 0 | 1. S. | 84. | 74.66 | 766 | 83. | 75. | 811 | 5 |
| 5 | 29.2 | | | 2 | 1. S.W. | 86. | 69.33 | 620 | 82. | 70.33 | 691 | 5 |
| 6 | 29.1 | 10, P.M. | | 2 | 1. S.W. | 84. | 72.33 | 702 | 82. | 63.33 | 566 | 6 |
| 7 | 29.3 | | 2, A.M. | 3 | 3. S.W. | 77. | 60.33 | 616 | 77. | 60.33 | 616 | 4 |
| 8 | 29.2 | 11, P.M. | | 5 | 3. N.W. | 75. | 61. | 654 | 74. | 60. | 657 | 10 |
| 9 | 29. | | 4, A.M. | 10 | 2. N. | 66. | 61.33 | 859 | 68. | 61. | 810 | 9 |
| 10 | | | | 1 | 3. N.E. | 67. | 53. | 653 | 64. | 54.66 | 737 | 5 |
| 11 | 29.2 | | | 2 | 2. N. | 67. | 53. | 653 | 69. | 59.66 | 764 | 2 |
| 12 | 29. | | | 8 | 2. N.W. | 70. | 56. | 656 | 73. | 61.33 | 695 | 1 |
| 13 | 29.3 | | | 10 | 1. N. | 70. | 51.33 | 558 | 68. | 56.33 | 696 | 1 |
| 14 | 29.1 | 5, A.M. | 9, A.M. | 6 | 1. S.W. | 74. | 72.66 | 971 | 71. | 69.66 | 969 | 3 |
| 15 | 29.3 | 5, A.M. | 11, A.M. | 10 | 1. N. | 68. | 63.33 | 860 | 67. | 62.33 | 858 | 3 |
| 16 | 29.3 | | | 0 | 1. S.W. | 66. | 52. | 649 | 63. | 53.66 | 737 | 6 |
| 17 | | | | 9 | 1. W. | 64. | 54.66 | 737 | 66. | 54.33 | 694 | 4 |
| 18 | 29.1 | 4, P.M. | 11, P.M. | 9 | 1. S. | 69. | 59.66 | 764 | 68. | 58.66 | 779 | 7 |
| 19 | 29. | | | 5 | 3. S.W. | 72. | 67.33 | 850 | 72. | 65. | 809 | 3 |
| 20 | 29.2 | | | 4 | 2. N.W. | 60. | 48.33 | 687 | 63. | 53.66 | 737 | 1 |
| 21 | 29.3 | | | 1 | 1. W. | 63. | 49. | 654 | 65. | 51. | 649 | 2 |
| 22 | 29.3 | | | 0 | 1. S.W. | 61. | 51.66 | 752 | 65. | 53.33 | 692 | 1 |
| 23 | 29.4 | | | 2 | 1. S.W. | 66. | 54.33 | 694 | 65. | 60.33 | 859 | 4 |
| 24 | | | | 0 | 1. S.W. | 72. | 55.33 | 619 | 66. | 59. | 811 | 1 |
| 25 | 29. | | | 5 | 1. W. | 76. | 57.33 | 560 | 70. | 63. | 809 | 0 |
| 26 | 29.1 | | | 0 | 1. S.W. | 75. | 68. | 809 | 72. | 62.66 | 761 | 1 |
| 27 | 29. | | | 0 | 1. S.W. | 74. | 67. | 809 | 73. | 66. | 809 | 0 |
| 28 | 29.2 | | | 0 | 1. W. | 70. | 58.33 | 699 | 67. | 57.66 | 765 | 0 |
| 29 | 29.5 | | | 1 | 1. N. | 64. | 47.33 | 609 | 66. | 61.33 | 859 | 0 |
| 30 | 29.5 | | | 0 | 1. S.W. | 61. | 49.33 | 688 | 64. | 52.33 | 692 | 0 |
| Aver., | 29.2 | | | 3.27 | | 71.47 | 59.78 | 710 | 70.40 | 61.05 | 790 | 89 |

EXPLANATION.— Clouds : 1 means that the sky is 1-10 covered ; 2, 2-10 covered, etc., etc. Winds : 1, a very light breeze ; 2, a light breeze ; 3, a fresh breeze ; 4, a strong wind.

